# Radiobiological restrictions and tolerance doses of repeated single-fraction hdr-irradiation of intersecting small liver volumes for recurrent hepatic metastases

**DOI:** 10.1186/1748-717X-5-44

**Published:** 2010-05-27

**Authors:** Ricarda Rühl, Lutz Lüdemann, Anna Czarnecka, Florian Streitparth, Max Seidensticker, Konrad Mohnike, Maciej Pech, Peter Wust, Jens Ricke

**Affiliations:** 1 Universitätsklinikum Magdeburg, Klinik für Radiologie und Nuklearmedizin, Otto-von-Guericke-Universität Magdeburg, Germany; 2 Charité Universitätsmedizin Berlin, Campus Virchow-Klinikum, Klinik für Strahlentherapie, Berlin, Germany; 3 Wroclaw Medical University, Department of Neuroradiology, Wroclaw, Poland

## Abstract

**Background:**

To assess radiobiological restrictions and tolerance doses as well as other toxic effects derived from repeated applications of single-fraction high dose rate irradiation of small liver volumes in clinical practice.

**Methods:**

Twenty patients with liver metastases were treated repeatedly (2 - 4 times) at identical or intersecting locations by CT-guided interstitial brachytherapy with varying time intervals. Magnetic resonance imaging using the hepatocyte selective contrast media Gd-BOPTA was performed before and after treatment to determine the volume of hepatocyte function loss (called pseudolesion), and the last acquired MRI data set was merged with the dose distributions of all administered brachytherapies. We calculated the BED (biologically equivalent dose for a single dose d = 2 Gy) for different α/β values (2, 3, 10, 20, 100) based on the linear-quadratic model and estimated the tolerance dose for liver parenchyma D_90 _as the BED exposing 90% of the pseudolesion in MRI.

**Results:**

The tolerance doses D_90 _after repeated brachytherapy sessions were found between 22 - 24 Gy and proved only slightly dependent on α/β in the clinically relevant range of α/β = 2 - 10 Gy. Variance analysis showed a significant dependency of D_90 _with respect to the intervals between the first irradiation and the MRI control (p < 0.05), and to the number of interventions. In addition, we observed a significant inverse correlation (p = 0.037) between D_90 _and the pseudolesion's volume. No symptoms of liver dysfunction or other toxic effects such as abscess formation occurred during the follow-up time, neither acute nor on the long-term.

**Conclusions:**

Inactivation of liver parenchyma occurs at a BED of approx. 22 - 24 Gy corresponding to a single dose of ~10 Gy (α/β ~ 5 Gy). This tolerance dose is consistent with the large potential to treat oligotopic and/or recurrent liver metastases by CT-guided HDR brachytherapy without radiation-induced liver disease (RILD). Repeated small volume irradiation may be applied safely within the limits of this study.

## Background

Irradiation of liver malignancies has been shown beneficial for patients with both primary and secondary intrahepatic tumors under specific oncological conditions, e.g. oligotopic metastases. Both stereotactic irradiation and image-guided brachytherapy have been described recently with promising results [1-6].

A dose-response relationship exists with an association between the delivery of a higher dose and improved clinical outcome [7] but since the liver is a radiosensitive organ there is an increasing risk of radiation-induced liver disease (RILD) when the whole organ is exposed to moderate doses, e.g. 30 Gy [8,9]. RILD, the most common liver toxicity after radiation therapy, is a clinical syndrome of anicteric hepatomegaly, ascites, and elevated liver enzymes occurring typically between 2 weeks to 3 months after completion of radiation therapy [10].

For this reason, external total liver irradiation plays a very limited role in the treatment of intrahepatic tumors. However, treatment of parts of the liver with higher radiation doses is possible without clinical consequences as long as an adequate volume of normal liver is spared.

Hepatic toxicity due to radiation therapy has been extensively investigated. Robertson et al. reported 12 of 26 patients with primary hepatobiliary cancers and measurable treatment-related toxicity. Doses ranged from 36 Gy (whole liver) to 72.6 Gy (focal liver). Two patients were diagnosed with nonfatal radiation hepatitis [11]. Cheng et al reported 12 out of 68 patients developing RILD after three-dimensional conformal radiotherapy (3D-CRT) of hepatocellular carcinoma with radiation portals designed to include the gross hepatic tumor on CT scan with 1.5-2 cm margins. No patient was given radiation to the whole liver. The mean dose was 50.2 Gy in daily fractions of 1.8-2 Gy [12]. Our own workgroup has previously published 2 papers on human hepatic dose tolerance after single small volume irradiation treatments employing the brachytherapy model and hepatocyte selective contrast agent to determine focal liver function loss. Whereas the mean dose threshold for lasting focal hepatic dysfunction was 15 Gy for all lesions. We found a considerable dose volume effect up to a threshold of 18 Gy favouring very small irradiation volumes [13,14]. However, no human in vivo data on dose tolerance or late toxic effects of repeated treatments of hepatic parenchyma is available today. The aim of the study described herein was to determine hepatic threshold doses for repeated small volume irradiation e.g. in case of tumor recurrence after previous radiation treatment of liver metastases, and to rule out the occurrence of any other toxic effects.

## Methods

### General methodology

**Figure 1:**
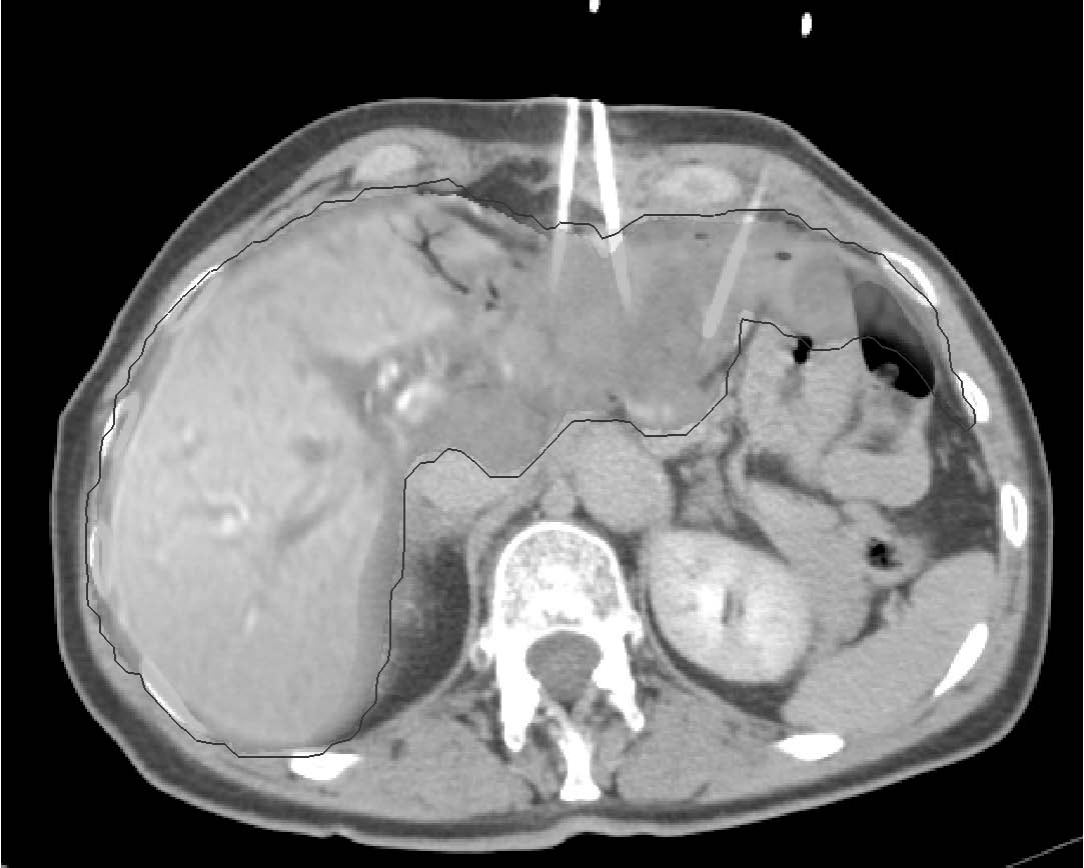
**Image-fusion: Contrast-enhanced computed tomography (CT) after CT-guided positioning of brachytherapy catheters (arrows) in a liver metastasis of a colorectal carcinoma, merged with the last magnetic resonance imaging of the liver acquired after all interventions (grey delineation)**. The hypointensity area shows the impairment of hepatocyte function in the left liver lobe.

**Figure 2:**
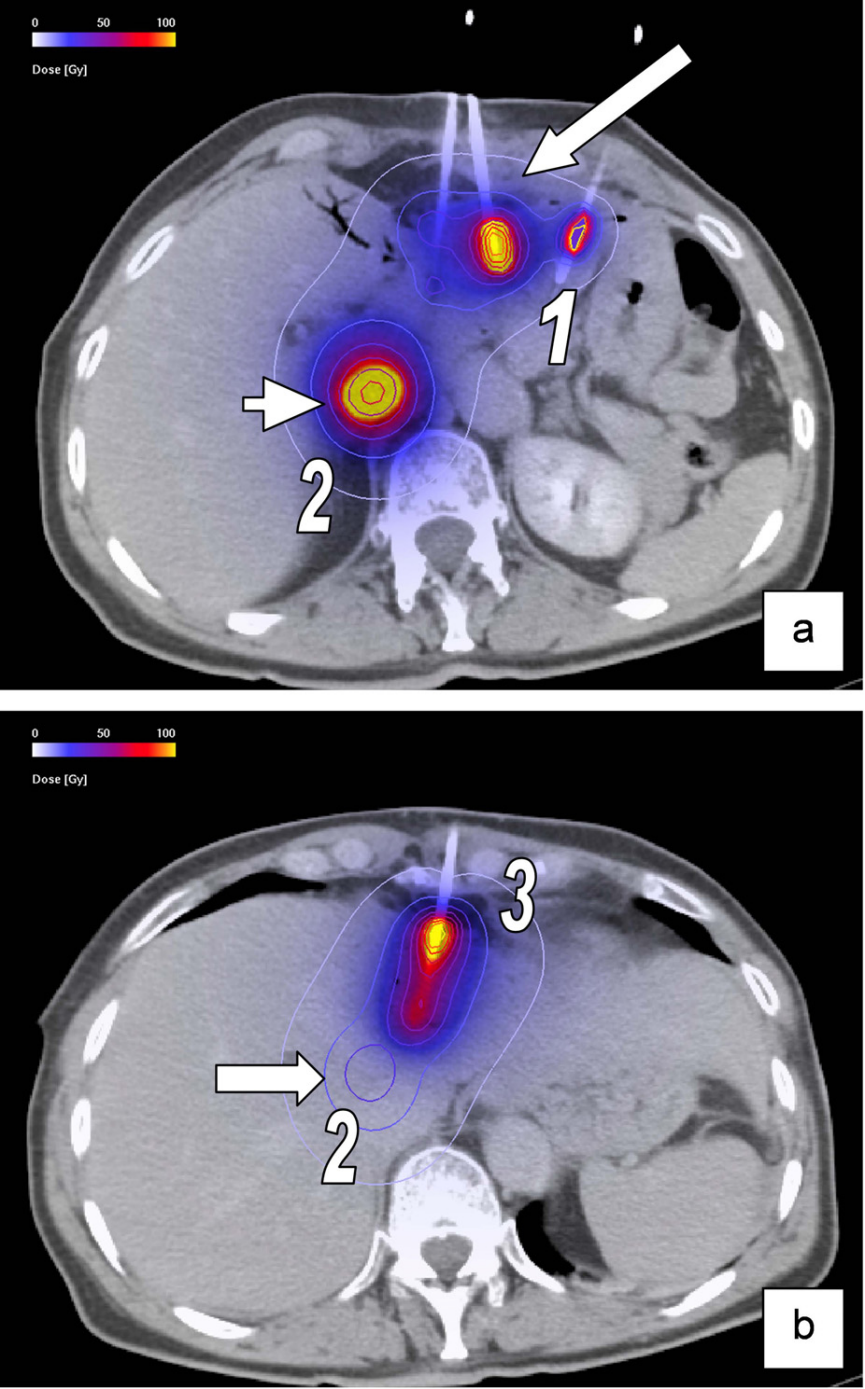
**Image fusion of CT-data set with the accumulated 3-D dosimetry of three different irradiation sessions**. (a) Contrast-enhanced CT after first (No.1) CT-guided positioning of brachytherapy catheters (long arrow) in metastases of a colorectal carcinoma. The short arrow shows the 3-D dosimetry of another lesion, irradiated in session No.2. (b) Contrast-enhanced CT after third intervention (No.3) in the same patient. The arrow shows the upper boundary of the 3-D dosimetry of the lesion irradiated in session No.2. Physical doses are shown in the colour map.

**Figure 3:**
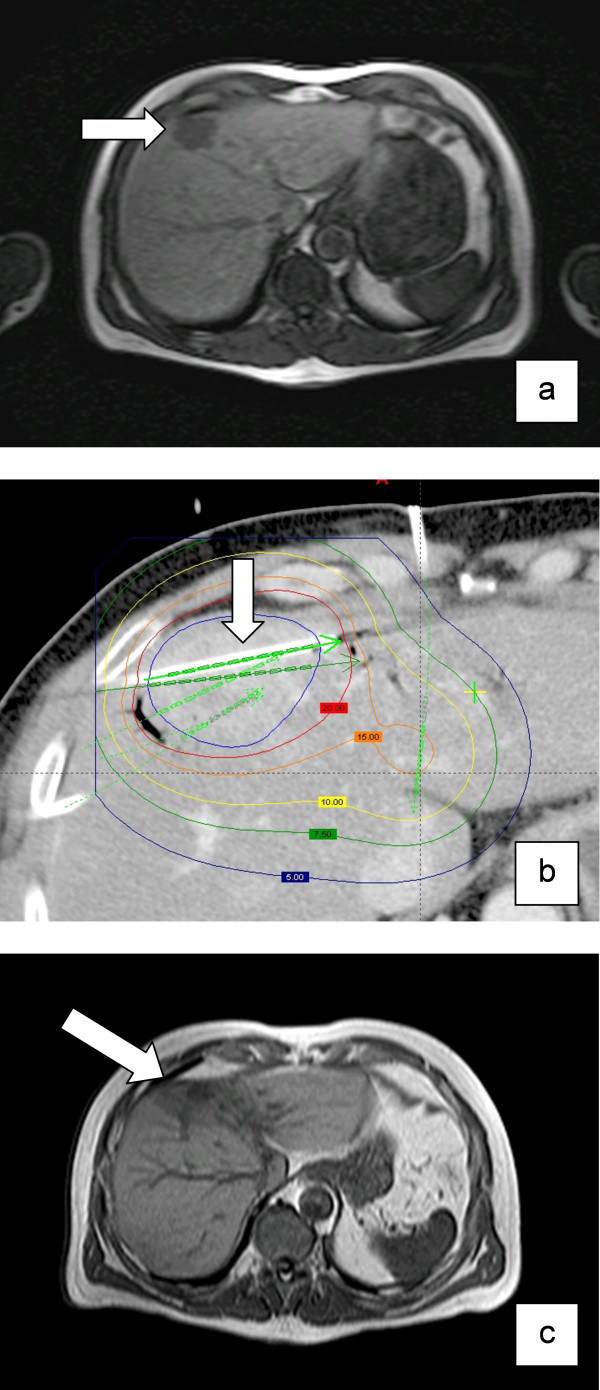
**Development of radiation injury of the liver after HDR brachytherapy: (a) Colorectal metastasis in liver segment IV (arrow), T1w-GRE 20 minutes after application of Gd-BOPTA**. (b) Contrast-enhanced planning-CT and dosimetry after insertion of brachytherapy catheters (arrow) in the metastasis. The coloured lines indicate different isodoses. Applicated dose at the tumor margin was 20 Gy. (c) MRI after 3 months with a decreased uptake (arrow) of Gd-BOPTA around the irradiated and shrunken metastasis (T1w-GRE 20 minutes after application of Gd-BOPTA).

**Figure 4:**
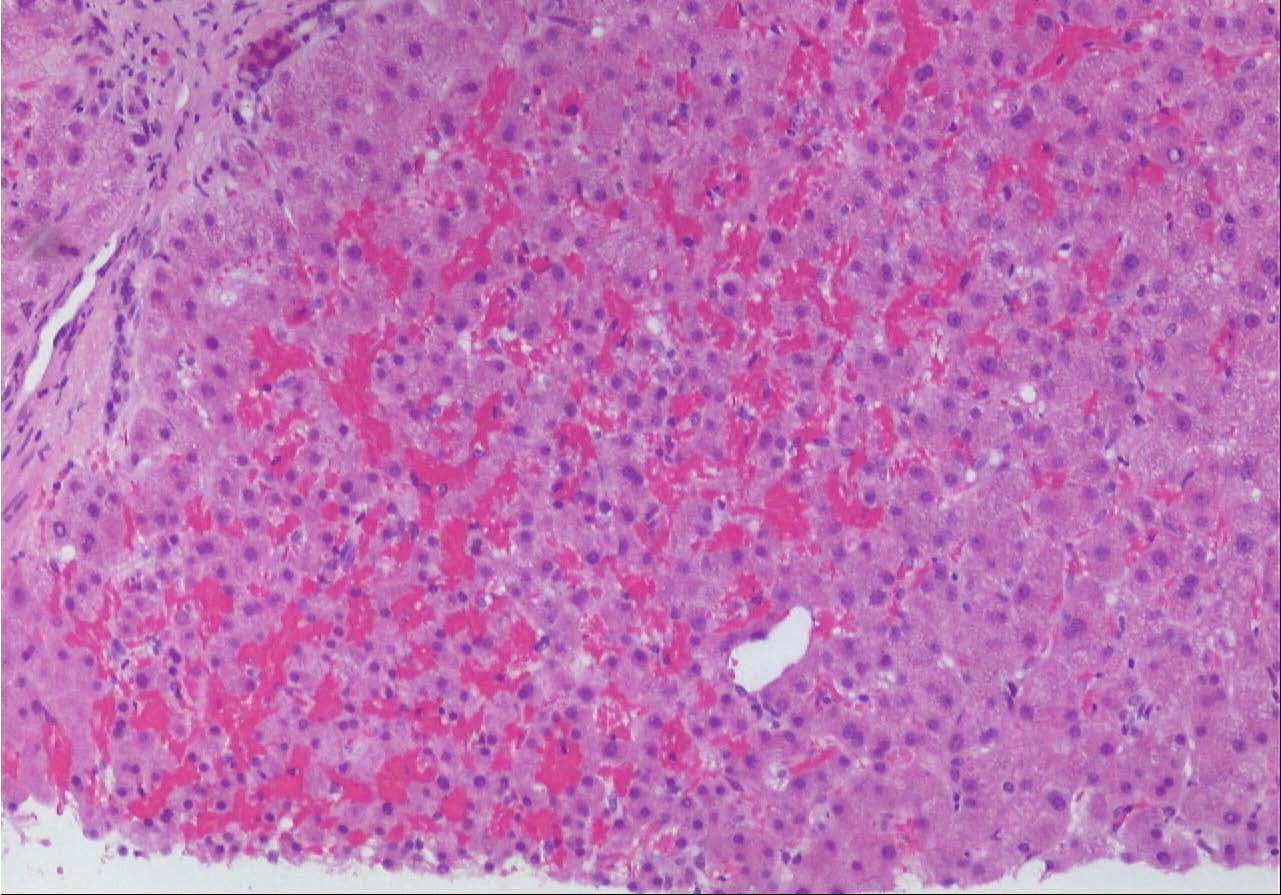
**Liver biopsy. Biopsy was performed to rule out a suspected local recurrence**. Tissue had been exposed to approximately 20 Gy two months ago. Heterogenous congestion of the sinusoids with beginning atrophy of liver cells. Hematoxylin-eosin, original magnification: ȕ100.

Patients eligible for this study had received at least 2 applications of computed tomography (CT)-guided brachytherapy of adjacent liver areas with intersecting dose distributions with time intervals of more than 4 weeks between radiation treatments. We sought to determine safety and clinical consequences of multiple applications of single-fraction irradiation of small liver volumes. We utilized a methodology previously described in a study on the tolerance dose of hepatic parenchyma after singular single-fraction HDR irradiation [13,14]. A fluoroscopy CT was used for catheter positioning and 3D-CT data sets are acquired for dose planning (Figure 1, 2). During follow-up to irradiation-therapy, magnetic resonance imaging (MRI) with the hepatocyte-directed contrast agent gadobenate dimeglumine (Gd-BOPTA) was selected to identify the function loss of liver parenchyma, hereinafter referred to as "cumulative pseudolesion". Gadobenate dimeglumine is an octadentate chelate of the paramagnetic ion gadolinium. Its kinetic properties resemble those of conventional iodinated contrast media and comprises a distribution phase and an elimination phase [15]. Studies have shown that this agent differs from other available gadolinium chelates in selectively being taken up only by functioning hepatocytes and excreted into the bile by the so-called canalicular multispecific organic anion transporter shared with bilirubin [15-17]. Changes in uptake of a hepatocyte specific contrast media illuminate the final path of the radiation injury, i.e. visualize areas of a dysfunctional hepatic system [18] (Figure 3). The histological appearance of radiation induced liver damage indicates that endothelial injury and subsequent obstruction of centrilobular venules and sinusoids are the key events in the pathogenesis of radiation injury of the liver. The pathological lesion resembles veno-occlusive disease [19-21] (Figure 4).

After image fusion, the isodose lines calculated for interstitial irradiation were projected onto the respective MRI scans. In the study described herein, we employed these techniques to assess the biologically equivalent tolerance dose of the irradiated volumes of liver parenchyma after repeated applications of single-fraction high-dose rate (HDR) brachytherapy. The LQ-model, established to predict late effects for different fractionation schemes, was adopted for the HDR-brachytherapy approach. The sensitivity of a tissue for a specific late effect was described by the critical dose α/β in Gy.

### Study population

We retrospectively analyzed the dose distributions of twenty patients. All patients received between two and four applications of CT-guided HDR brachytherapy either of the same liver lesion or in close proximity due to local tumor recurrence or growth of a satellite lesion. Written informed consent was obtained from all patients.

The patient population comprised of 10 men and 10 women; mean age was 64 years (51-84 years). Primary malignancies were colorectal carcinoma (n = 18), cholangiocellular carcinoma (n = 1) and breast carcinoma (n = 1). Karnofsky performance score was higher than 80%. Nineteen patients had received systemic anticancer treatments before brachytherapy, terminated at least 4 weeks before tumor ablation. Some of these drugs cause specific toxic effects in the liver as steatosis and steatohepatis, hyperbilirubinaemia and vascular changes, sinusoidal obstruction or dilatation syndrome. Patients received in particular irinotecan (n = 8), fluorouracil (n = 15), capecitabine (n = 2), oxaliplatin (n = 5), epirubicine (n = 1) and gemcitabine (n = 1) before intervention. In between the interventions and during postinterventional surveillance 7 patients received again irinotecan, 5 patients received fluorouracil and oxaliplatine respectively, capecitabine (n = 3), avastine (n = 2) and UFT (n = 1).

Treatment was carried out consecutively without selection or randomisation. There was a minimum interval of 4 weeks and a maximum of 14 months between sequential applications (table 1). The decision to treat or re-treat any lesion was taken individually following oncological considerations.

**Table 1:** The extent of the irradiation effects ("cumulative pseudolesion" in cm^3^) and calculated D_90 _(for α/β = 2 Gy) with respect to the minimal prescribed dose inside of the clinical target volume (CTV) in the different irradiation sessions.

Pat.	**D**_**90 (2 Gy)**_	Cum. Pseudo-lesion Volume (cm^3^)	Fractions (n)	Interval between inter-ventions (months)	Interval between first irradiation & last MRI (months)	Interval between last irradiation & last MRI (months)	Minimal prescribed dose inside CTV (Gy)	Liver Volume (cm^3^)
1	18.80	289	3	3/3	17	11	20/20/20	1960

2	16.20	327	2	7	14	7	25/15	2184

3	35.20	135	4	7/1/8	31	3	15/20/20/20	1123

4	25.30	172	3	4/3	18	11	20/20/15	1548

5	17.20	710	2	12	5	4	15/15	2077

6	25.15	381	2	13	13	12	15/20	1357

7	16.40	239	2	7	18	11	25/15	1847

8	14.35	803	2	6	13	7	15/20	1463

9	31.90	92	4	4/4/8	24	7	15/15/20/20	1424

10	23.65	425	2	14	18	4	20/15	2054

11	28.35	178	2	5	10	4	15/20	1662

12	21.64	668	3	4/6	16	6	20/15/20	2165

13	21.70	207	2	10	12	3	15/15	1439

14	15.90	529	3	1/5	9	4	15/15/15	1441

15	16.30	238	2	11	14	3	20/15	2025

16	21.75	244	3	1/7	10	3	20/20/15	1604

17	33.20	476	4	1/10/1	19	9	25/15/25/25	1951

18	20.55	227	2	1	5	4	20/20	1387

19	14.90	319	2	11	10	8	15/15	1428

20	29.60	75	2	9	12	3	20/20	1246

								

Mean	22.40	336.7	3	5	14	6	20	1669.16

The different time intervals between the interventions and the different time intervals between the interventions and acquisition of MRI examinations are shown.

**Table 2:** The D90 for impairment of hepatocyte function ("cumulative pseudolesion") calculated for different α/β-values (2, 3, 10, 20, 100).

D_90 _(Gy) for impairment of hepatocyte function for different α/β-values					
Patient	α/β = 2	α/β = 3	α/β = 10	α/β = 20	α/β = 100
1	18.80	19.00	19.99	20.90	23.60

2	16.20	16.40	16.65	17.30	18.80

3	35.20	35.70	36.50	36.90	38.10

4	25.30	25.40	26.20	27.00	29.60

5	17.20	17.30	18.00	18.65	20.45

6	25.15	25.20	25.65	26.15	27.85

7	16.40	16.60	17.50	18.45	20.70

8	14.35	14.45	15.20	15.87	17.55

9	31.90	32.60	33.40	34.00	36.80

10	23.65	23.80	24.75	25.78	28.80

11	28.35	28.40	29.90	30.90	34.70

12	21.64	21.70	22.65	23.48	26.10

13	21.70	21.80	22.45	23.10	25.00

14	15.90	16.10	17.10	17.90	20.10

15	16.30	16.40	16.95	17.70	19.45

16	21.75	21.90	22.10	22.90	25.20

17	33.20	34.10	34.90	35.60	37.10

18	20.55	20.60	20.85	21.35	22.50

19	14.90	15.10	16.00	16.80	19.00

20	29.60	29.90	30.00	30.80	31.90

					

Mean	22.40	22.62	23.34	24.08	26.17

Stand. deviation	6.47	6.62	6.64	6.61	6.68

Only patients who underwent more than one application of CT-guided HDR-brachytherapy with intersecting dose distributions were included. Normal liver function based on laboratory parameters as well as clinical examination was acquired before CT-guided brachytherapy.

We excluded patients with any clinical or laboratory sign of liver function degradation before therapy.

### Interventional technique and irradiation

The technique of CT-guided brachytherapy has been described in detail elsewhere [22]. Positioning of the brachytherapy applicators was performed with a fluoroscopy CT (Siemens, Erlangen, Germany). After catheter placement a spiral CT of the liver (slice thickness: 5 mm, increment: 5 mm), enhanced by i.v. application of iodide contrast media (100 mL Ultravist 370, flow: 1 mL/s; start delay: 80s), was acquired using the breath-hold technique for treatment planning purposes.

The HDR afterloading system (GammaMed, Varian, Charlottesville,VA) used a ^192^Iridium source of 10Ci. The source diameter was <1 mm and dwell positions were located every 5 mm. Dwell times were corrected automatically according to the actual source strength.

### Treatment planning and dosimetry analysis

Treatment planning employed BrachyVision (Varian Medical Systems, Palo Alto, CA). A radiation oncologist and radiologist jointly performed the planning process, i.e. delineation of the clinical target volume CTV (gross tumor volume GTV plus safety margin of a few mm) according to clinical considerations. The prescribed dose to enclose the CTV ranged from 15 to 25 Gy (mean 20 Gy, average 18.27 Gy) (table 1). In organs at risk (intestine, stomach) D_1 ml _was prescribed to be < 15 Gy. The volume dose to the liver (D_15 Gy_, D_10 Gy_, D_7 Gy_) was kept as small as reasonable.

All applications were performed as a single dose employment.

### Follow-Up

MRI examinations were performed in 20 patients 1 day before, 3 days, 6 weeks and following every 12 weeks after irradiation. The MRI protocol was comprised of the following sequences: T2-w breathing-triggered UTSE (TE/TR 90/2,100 ms), T1-w breath-hold gradient echo (GRE) (TE/TR 5/30 ms, flip angle 30°) precontrast, 20 s and 2 h post i.v. application of 15 mL Gd-BOPTA (Multihance; Bracco, Princeton, NJ). The slice thickness was 8 mm, acquired in interleafed mode with no gap applied.

At the same control dates we also assessed the following laboratory parameters: bilirubin, aspartate aminotransferase, alanine aminotransferase, alkaline phosphatase, albumin, ammonia and C-reactive protein to assess treatment-related toxicity using Radiation Therapy Oncology Group (RTOG) toxicity score.

### Image registration

All 3D-dosimetry data calculated by BrachyVision during all CT-guided brachytherapies were merged with the last MRI-data set which had been acquired during a period of ≤12 months after the last intervention. All data were processed by anisoscalar image registration (Figure. 1, 2). By reducing the images to contain liver parenchyma and a small surrounding margin (approx. 1 cm), anisotropic image fusion was sufficient to achieve accuracy better than 5 mm for the liver surface and prominent anatomic structures (e.g. large vessels) [14].

The registration routine of the algorithm was based on normalized mutual information and has been described by Studholme et al. [23]. We employed a modified independent implementation of this algorithm within the 3D visualization software Amira version 3.1 (Mercury Computer Systems, Inc, San Diego, USA). The accuracy of the implemented algorithm was verified by Rohlfing et al. [24].

### Quantitative analysis

For every patient, we calculated the cumulative biologically equivalent dose BED in every voxel over the whole liver for different α/β-values (2, 3, 10, 20, 100) by the following equation [25]:(1)

where D_K _is the dose per voxel deposited during the intervention k = 1,...,n and D_tot _is the BED per voxel with respect to conventional fractionation.

Every lesion was the result of repetitive single interventions with different dose distributions. On the T1-w late Gd-BOPTA enhanced images of the last MRI acquired after the last therapy, two experienced GI-radiologists evaluated the fused images of MRI and dose distributions by delineating the border of hypointensity in the irradiated liver area in consensus. In liver regions with no detectable uptake of Gd-BOPTA (hypointensity) we assumed radiation-induced damage of liver tissue [13,14].

**Figure 5:**
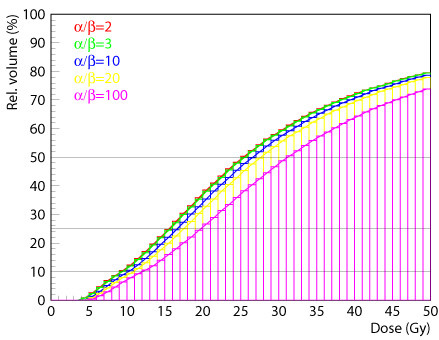
**Based on the total 3D-D_tot_-data set, Amira software calculated dose-volume histograms for all different α/β-values**.

Based on the total 3D D_90_-data set, Amira software calculated the dose-volume histograms for a set of α/β values (2, 3, 10, 20, 100) (Figure 5) to determine the D_90 _(α/β) for every cumulative pseudolesion, i.e. the BED exposing 90% of the pseudolesion in MRI.

### Statistical analysis

Standard Pearson correlation coefficients were determined to perform an univariate correlation analysis with following variables used: D_90 _(for α/β = 2), number of interventions, interval between first irradiation and last MRI, interval between last irradiation and last MRI, mean interval between several interventions and volume of the cumulative pseudolesion. Kendall's W-test for related samples was used to test the difference with respect to various α/β-values.

We compared 2 groups of patients with pseudolesion volumes of ≤200 mL (n = 5) or >200 mL (n = 15). The comparison between the D_90(2 Gy) _of the two groups was performed by using an unpaired *t- *test.

Analyses were performed using the Statistical Package for the Social Sciences, version 13.0.0 (SPSS for Windows, Chicago, Illinois, USA). A p-value < 0.05 was considered statistically significant.

## Results

The volumes of radio-affected liver tissue (pseudolesion) are presented in Table 1 in dependency on the time intervals between the interventions and the time intervals from interventions to MRI examinations. The lesion size overall ranged from 75 cm^3 ^to 803 cm^3 ^(median 266.5 cm^3^), the whole liver volume ranged from 1123 cm^3 ^to 2184 cm^3 ^(median 1576 cm^3^). The time interval from the first brachytherapy to the last MRI ranged from 5 to 31 months (median 13.5 months), and from the last brachytherapy to the last MRI from 3 to 12 months (median 5 months), respectively. The MRI-data we used was the latest MRI acquired during a period of median 5 months after the last intervention.

**Figure 6:**
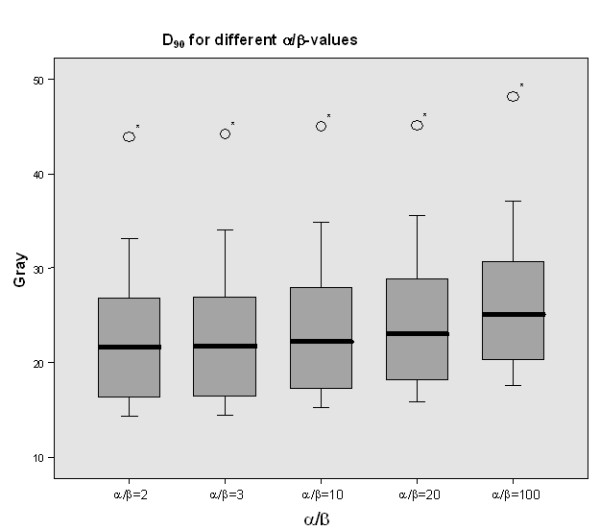
**Mean D_90 _for different α/β-values (2, 3, 10, 20, 100) of all patients**. Statistically there was a significant difference in the D_90 _results but the differences for clinical α/β-values were less than 1 Gy and therefore of no clinical. The star (*) represents one outlier in the series of calculation.

Table 2 shows the calculated D_90 _covering the inactivated liver tissue corresponding to variable α/β values. The mean tolerance doses ranged from 22.40 Gy for α/β = 2 to 23.34 Gy for α/β = 10, and 24.08 Gy for α/β = 20 to 26.17 Gy for α/β = 100, respectively. The differences in the D_90 _were statistically significant, but the differences for clinical relevant α/β-values between 2 and 10 were smaller than 1 Gy (table 2, Figure 5, 6).

The D_90 _(for α/β = 2) and the interval between the first irradiation and the last MRI correlated significantly (p = 0.005) as a result of ongoing repair or regeneration. A correlation was also shown between the D_90 _and the number of interventions, likely as a result of better recovery in case of a smaller number of irradiation treatments (p = 0.004) (table 3). There was a trend for a positive correlation (p = 0.092) between D_90 _and the mean interval between the whole series of several interventions. The time interval between repeated interventions did increase the D_90(2 Gy)_, indicating that the time available for regeneration influenced the dose tolerance. Conversely, a significant inverse relationship (p = 0,037) between the volume of the pseudolesion and D_90 _was found (table 3).

**Table 3:** D90 (for α/β = 2) tested against various variables for correlation analysis.

PEARSON CORRELATION	D_90 _(α/β = 2)
Interventions (n)	p = 0.004

Cumulative Pseudolesion (cm^3^)	p = 0.037

Interval: first irradiation - MRT (months)	p = 0.005

Interval: last irradiation - MRT (months)	p = 0.745

Mean interval between several interventions (ΣT/n)	p = 0.092

The D90 (for α/β = 2) correlated significantly positive with the number of interventions and the interval between the first irradiation and the last MRI (p < 0.05). There was a significant negative correlation between the D90 (for α/β = 2) and the volume of the cumulative pseudolesion (p = 0,037). There was a trend for a positive correlation (p = 0.092) between the D90 (for α/β = 2) and the mean interval between several interventions (ΣT/n).

After definition of three groups with different pseudolesion volumes (1: up to 199 mL (30.1 Gy), 2: 200-399 mL (19.1 Gy) and 3: ≥400 mL (21 Gy)), the Bonferroni-test revealed a significant difference for the D_90(2 Gy) _between group 1 and both other groups, while there was no difference between group 2 and 3. However, there was a cut off at 200 mL and the comparison of the two groups regarding patients with different pseudolesion volumes showed a difference between the D_90(2 Gy) _(30.1 Gy vs. 19.1 Gy) (p = 0.01) (table 4).

**Table 4:** The comparison between the two groups of different cumulative pseudolesion volumes (≤200 mL; >200 mL) showed a statistically significant difference in the D90 (α/β = 2).

Critical irradiation volume	D_90 _(α/β = 2)
Cum. Pseudolesion ≤200 mL (n = 5)	30.1 Gy

Cum. Pseudolesion >200 mL (n = 15)	19.1 Gy

t-test	p = 0.01

The statistically significant level was set at p-value ≤ 0.05.

There was no significant correlation with all the other variables tested: a) the interval from last irradiation to MRI did not correlate with the D_90(2 Gy) _(p = 0.834); b) the intervals between repeated irradiations and MRI did not correlate with the mean interval between all interventions. Next to that, the number of interventions did not negatively influence hepatocyte function recovery at specific dose levels.

All patients included in this study demonstrated normal liver function parameters before CT-guided brachytherapy. There was no Grade 2 or above hematologic toxicity according RTOG toxicity scale. No patient developed symptoms of acute or late chronic liver dysfunction in between the interventions or during follow up, which could be related to irradiation.

## Discussion

The tolerance doses of the entire liver or large portions of the liver to external irradiation have been described previously in the literature. A TD_5/5 _of 30 Gy is given for the whole liver, while one-third to two-third of the liver tolerate higher doses of 35 Gy to 50 Gy, respectively [19,26]. Small volume effects have been described for both stereotactic radiation as well as CT-guided brachytherapy treatments [1,5,14]. Promising results with sustained local control rates have been achieved.

Ricke et al. reported a median survival of 23.4 months after image-guided high dose rate brachytherapy (minimal tumor-enclosing doses of 15 Gy, 20 Gy, or 25 Gy as D100) of seventy-three patients with 199 colorectal liver metastases [5].

Lee et al. showed a median survival of 17.6 months in sixty-eight patients with inoperable liver metastases being treated with individualized stereotactic body radiotherapy (SBRT) with a median SBRT dose of 41.8 Gy in six fractions over two weeks [2]. No RILD or other grade 3 - 5 liver toxicity was seen. Also Rusthoven et al. demonstrated in a multi-institutional trial with forty-seven patients (with one to three liver metastases) that high-dose liver SBRT is safe and effective with a median survival of 20.5 months with only one patient presenting with grade 3 toxicity [6]. Localablative irradiation therapies of liver malignancies are of great value especially for patients who are not suitable for surgical interventions.

In this study we sought to determine threshold doses for hepatic dysfunction as well as toxic effects e.g. after repeated irradiation treatments of liver metastases due to local failure. As a result we found that repeated sessions of high dose rate, single fraction irradiation at very high dose levels targeting identical or intersecting liver volumes were safe.

In our trial we did not observe any acute or long term toxicity despite hepatic dysfunction in areas of high dose accumulation.

One relevant factor for the tolerance to irradiation is the critical single dose α/β in Gy, which describes the sensitivity to the dose per fraction and/or dose rate of a particular tissue, either tumor or organ. The ratio α/β describes the initial form of the curvature of the underlying cell survival curves [27-29]. Small α/β-ratios are associated with a broader shoulder of the dose-response curve indicating a large dependency of the radiation effect on the dose per fraction, while large values of α/β indicate only minor fractionation sensitivity. These individual endpoints for specific tissues were first described in animal studies [30] and have been confirmed by numerous clinical data [29].

In our study we calculated the D_90 _of the pseudolesions for different α/β-values (2, 3, 10, 20 and 100) and did not find clinically relevant differences. The BED (biologically equivalent dose) causing injury to 90% of liver parenchyma was approximately 23 Gy (table 2). Obviously, the superposition of dose distributions with large gradients reduces the dependency on α/β.

Time factors (describing repair kinetics) had no or negligible influence in our study since the interval between two applications (between 4 weeks and 14 months, mean 5 months, table 1) was long enough to ensure sufficient repair [31,32]. We additionally propose that the dose rate variability as a result of the distance relative to the catheters had no relevant influence on repair capacity.

However, we observed a trend to a correlation of the D_90 _and the time intervals between the interventions as a result of regeneration or recovery of the irradiated liver tissue. This is in accordance with 2 previous trials performed by our own group, where the greatest volume of function loss after single applications of CT-guided HDR brachytherapy occurred after 6 weeks to 3 months [13,14]. Six months after irradiation, the volume with dysfunctional liver tissue had decreased significantly. We deduced a mean tolerance dose for irreversible damage above a dose level of 15 Gy (D_90_) applied as a single radiation dose. At a dose exposure between 10 and 15 Gy, hepatic dysfunction proved to be reversible [13]. These results are somewhat contrary to a trial performed by Lawrence et al. who demonstrated just minor recovery of liver cell plates after six months and up to 6 years [19].

In our present trial, MRI was acquired between 3 and 31 months (median 10 months) after irradiation and we expected liver regeneration in the irradiated areas between 10 - 18 Gy. Nevertheless, we still found a significant dependency between the D_90 _and the time interval from the first irradiation to the last MRI. In addition, we found a trend to a correlation of the D_90 _and the time intervals between the different irradiation sessions (table 3). Therefore, a long-term regeneration potential probably exists. With respect to the clinical endpoint liver failure we wish to add that even after multiple applications of HDR-brachytherapy in adjacent liver areas the (cumulative) volume of radiation injury did never exceed 803 mL (mean 336.7 mL). We therefore never reached a treatment volume critical for the overall liver function, i.e. >60 - 70% of the whole liver volume (mean 1669.16 mL).

On the other hand an inverse correlation between the overexposed (i.e. damaged) volume size and the equivalent tolerance dose (isoeffect-isodose for the impairment of hepatocyte function) was found. A volume threshold was found at 200 mL (table 4). For volumes <200 mL the tolerance dose increased up to 30 Gy. These results are in line with the previously published dose-volume effect of hepatic repair [14]. The tolerance dose of D_90 _(single dose) as determined in a previous study for a single HDR application was ~ 15 Gy [13]. A re-calculation of the BED from 15 Gy (single) for different α/β to conventional fractionation would result in a BED (α/β = 3) of 54 Gy, BED (α/β = 5) of 42 Gy, or BED (α/β = 10) of 31 Gy. In comparison, the tolerated D_90 _(2 Gy) of BED ~ 23 Gy in our study proved to be surprisingly low and suggests in particular that α/β for liver tissue might be higher than 5 Gy. The first (and most important) reason for this contradictory result probably is the small liver volume exposed to a single high dose in the referenced trial [13]. Under these circumstances a higher potential for regeneration might exist. For larger volumes (>200 mL) and repeated HDR applications, the tolerance doses were even below the limits for whole liver irradiation of approximately 30 Gy as described in the literature. However it has to be mentioned that most of the patients have been treated with potentially hepatotoxic cytotoxics or new biological agents before or between the interventions, which may cause specific toxic effects in the liver and potentially lead to a reduced tolerance of the liver although most adverse reactions are idiosyncratic and are due to individual patient differences in susceptibility to drug-induced liver injury or inability to recover from the injury. Most patients tolerate the agent, or can adapt to it [33]. Clinically one-third or even two-thirds of the liver can be inactivated with no symptomatic liver function degradation. This is not clearly stated in the Emami report in 1991, which established baseline partial liver tolerances [26]. Dawson et al. further adjusted the Lyman model parameters in 2002 and derived the TD5/5 (in 1.5 Gy BID) for 1/3 of the liver volume = 107 Gy (~94 Gy in 2 Gy/fx), for 2/3 = 54 Gy (~48 Gy in 2 Gy/fx). They calculated a 5% risk of RILD for whole liver radiation therapy (3/3) with 32 Gy in 2 Gy/fx [7].

In our study the tolerance doses of liver parenchyma fell in the range of 22 - 24 Gy (conventional fractionation), which is clearly below the data in the literature. This might be a result of chemotherapy pretreatments in almost all our patients. However, even a BED of 22 - 24 Gy as determined in this study implies large clinical potential for irradiation of liver metastases if the hepatic radiation injury is limited to moderate volumes (table 1). Furthermore, we did not find clinically relevant late toxicity in any patient undergoing multiple applications of high-dose-rate brachytherapy. In none of our imaging studies fibrotic changes or considerable hypertrophy of the uninvolved liver was documented. All patients demonstrated normal liver function parameters before and after CT-guided brachytherapy.

## Conclusion

We conclude that repeated high dose rate single fraction irradiation of intersecting liver volumes is safe. Very high tumor doses and repeated applications of brachytherapy and potentially stereotactic irradiation are possible for liver metastases treatments without an increased risk of liver failure. In our opinion caution is warranted in whole liver irradiation applying external techniques.

Our data suggests that the tolerance dose in a pretreated liver might not be < 30 Gy using fractions of 2 Gy as stated in the literature but as low as 22 - 24 Gy.

## Competing interests

The authors declare that there is no actual or potential conflicts of interest, sources of financial support, corporate involvement, patent holdings, etc. for each author to report.

## Authors' contributions

RR participated in the study's design and coordination, performed acquisition of data and the analysis of images, performed the statistical analysis and draft the manuscript. LL participated in the study's design, performed the statistical analysis and helped to draft the manuscript. AC and FS performed acquisition of data and the analysis of images. MS and KM participated in the study's coordination and helped to draft the manuscript. JR and PW conceived of the study, and participated in its design and coordination and helped to draft the manuscript. All authors read and approved the final manuscript.
